# Does Evidence Exist to Blunt Inflammatory Response by Nutraceutical Supplementation during COVID-19 Pandemic? An Overview of Systematic Reviews of Vitamin D, Vitamin C, Melatonin, and Zinc

**DOI:** 10.3390/nu13041261

**Published:** 2021-04-12

**Authors:** Salvatore Corrao, Raffaella Mallaci Bocchio, Marika Lo Monaco, Giuseppe Natoli, Attilio Cavezzi, Emidio Troiani, Christiano Argano

**Affiliations:** 1 Department of Health Promotion Sciences, Maternal and Infant Care, Internal Medicine and Medical Specialties, [PROMISE], University of Palermo, 90127 Palermo, Italy; 2 COVID Unit, Department of Internal Medicine, National Relevance and High Specialization Hospital Trust ARNAS Civico, Di Cristina, Benfratelli, 90127 Palermo, Italy; raffaellamallacibocchio@gmail.com (R.M.B.); marika.lomonaco@hotmail.it (M.L.M.); peppenatoli@gmail.com (G.N.); chargano@yahoo.it (C.A.); 3 Eurocenter Venalinfa, 63074 San Benedetto del Tronto, Italy; info@cavezzi.it; 4 Cardiology Unit, State Hospital, Social Security Institute, 20, 47893 Cailungo, San Marino; emidio.troiani@iss.sm

**Keywords:** COVID-19, SARS-CoV-2, overview, systematic review, vitamin D, vitamin C, melatonin, zinc, inflammation, nutraceuticals

## Abstract

More than one year has passed since the first cases of coronavirus disease 2019 (COVID-19) caused by severe acute respiratory syndrome (SARS)-CoV-2 coronavirus were reported in Wuhan (China), rapidly evolving into a global pandemic. This infectious disease has become a major public health challenge in the world. Unfortunately, to date, no specific antivirals have been proven to be effective against COVID-19, and although a few vaccines are available, the mortality rate is not decreasing but is still increasing. One therapeutic strategy has been focused on infection prevention and control measures. In this regard, the use of nutraceutical supports may play a role against some aspect of the infection, particularly the inflammatory state and the immune system function of patients, thus representing a strategy to control the worst outcomes of this pandemic. For this reason, we performed an overview including meta-analyses and systematic reviews to assess the association among melatonin, vitamin C, vitamin D, zinc supplementation and inflammatory markers using three databases, namely, MEDLINE, PubMed Central and the Cochrane Library of Systematic Reviews. According to the evidence available, an intake of 50,000 IU/month of vitamin D showed efficacy in CRP. An amount of 1 to 2 g per day of vitamin C demonstrated efficacy both in CRP and endothelial function, and a dosage of melatonin ranging from 5 to 25 mg /day showed good evidence of efficacy in CRP, TNF and IL6. A dose of 50 mg/day of elemental zinc supplementation showed positive results in CRP. Based on the data reported in this review, the public health system could consider whether it is possible to supplement the current limited preventive measures through targeted nutraceutical large-scale administration.

## 1. Introduction

The coronavirus disease 2019 (COVID-19), caused by SARS-CoV-2, has rapidly spread worldwide. The clinical spectrum of SARS-CoV-2 severity may range from asymptomatic to severe conditions, including acute respiratory distress syndrome and sometimes leading to multiorgan failure. For most people, a piece of important information is to know how to strengthen the immune system to prevent SARS-CoV-2 infection to avoid the next waves of the deadly COVID-19 pandemic [1]. It is well known that the presence of chronic diseases may exacerbate the inflammatory response induced by coronavirus disease, increasing the risk of severe disease and mortality. In this respect, systemic inflammation developing in people with non-communicable diseases, such as diabetes, tends to exacerbate the respiratory symptoms of infection [2]. Additionally, obesity and central adiposity represent important risk factors for complications of COVID-19, especially in patients with impaired heart and lung function [3]. Furthermore, the vascular damage frequent in diabetic patients and people with hypertension increases the risk of these individuals being affected by COVID-19 thrombotic complications. The latest report issued by WHO mentioned more than 96 million confirmed cases and more than two million deaths worldwide [4]. To reduce the risk of transmission of SARS-CoV-2, several preventive measures have been advised for general public health, including hand and respiratory hygiene and safe food practices (concerning raw animal products) to reduce the risk of transmission of SARS-CoV-2 [5]. The use of both a correct life-style and nutraceutical supports of proven efficacy may play a role against some aspect of the infection, thus representing a strategy to control this epidemic. In the current situation, it is urgent to propose preventive health measures to reduce the risk of COVID-19 infection in addition to an adequate vaccine diffusion and/or effective antiviral drugs. In the COVID-19 pandemic, the importance of adequate nutrition and eating habits has been widely emphasized, not only to reduce the impact of the widely diffused non-communicable diseases that may cause more severe infections (e.g., diabetes and obesity) but also as a way to regulate the inflammatory state of patients. In fact, underestimating the importance of nutrition for COVID-19 patients will significantly affect the prognosis of these subjects [6]. Vitamins C and D and trace elements, including zinc [7], may play a fundamental role in disease susceptibility and maintaining immune system function [8]. In fact, COVID-19 is characterized by high levels of inflammatory markers, including C-reactive protein (CRP) and increased levels of inflammatory cytokines and chemokines [9,10]. Any nutritional/nutraceutical approach potentially useful to regulate immune function may consequently be beneficial both in the early phase, when an adequate immune reaction is fundamental, and in case of later cytokine storm, when the hyper-reactive immunity may be detrimental. This overview aims to analyze the current knowledge from systematic reviews of the relationship between nutraceutical supports and the reduction in inflammatory response to formulate clinical recommendations and highlight directions for future research during the COVID-19 pandemic. The influence of nutraceutical compounds on inflammatory markers will be addressed.

## 2. Materials and Methods

### 2.1. Eligibility Criteria

All meta-analyses and systematic reviews (SRs) regarding the association among melatonin, vitamin C, vitamin D, zinc supplementation and inflammatory markers were eligible for this overview.

### 2.2. Search Methods

On 22 September 2020, at 10:06 a.m. (GMT -5, Bethesta, MA, USA), a literature search was performed, regarding MEDLINE, PubMed Central and the Cochrane Library of Systematic Reviews (CLSR) and using the following search strings.

#### 2.2.1. Melatonin

(“melatonin” [MeSH Terms] OR “melatonin” [All Fields] OR “melatonin s” [All Fields] OR “melatonin” [All Fields] OR “melatonins” [ All Fields]) AND (“inflammatories” [All Fields] OR “inflammatory” [All Fields] OR (“inflammation” [MeSH Terms] OR “inflammation” [All Fields] OR “inflammations” [All Fields] OR “inflammation s “[All Fields]) OR” TNF “[All Fields] OR (“ interleukine “[All Fields] OR” interleukines “[All Fields] OR” interleukins “[MeSH Terms] OR” interleukins “[All Fields] OR” interleukin “[All Fields]) OR (“ cytokin “[All Fields] OR” cytokine s “[All Fields] OR” cytokines “[MeSH Terms] OR” cytokines “[All Fields] OR” cytokine “[All Fields] OR” cytokinic “[All Fields] OR” cytokins “[All Fields])). 

#### 2.2.2. Vitamin C

(“ascorbic acid” [MeSH Terms] OR (“ascorbic” [All Fields] AND “acid” [All Fields]) OR “ascorbic acid” [All Fields] OR “ vitamin c “[All Fields] OR (“ ascorbate “[All Fields] OR” ascorbates “[All Fields] OR” ascorbic “[All Fields]) OR (“ ascorbate “[All Fields] OR” ascorbates “[All Fields] OR “ascorbic” [All Fields])) AND (“inflammation” [MeSH Terms] OR “inflammation” [All Fields] OR “inflammations” [All Fields] OR “inflammation s” [All Fields] OR (“inflammatories” [ All Fields] OR “inflammatory” [All Fields]) OR “TNF” [All Fields] OR (“interleukine” [All Fields] OR “interleukines” [All Fields] OR “interleukins” [MeSH Terms] OR “interleukins” [ All Fields] OR “interleukin” [All Fields]) OR (“cytokin” [All Fields] OR “cytokine s” [All Fields] OR “cytokines” [MeSH Terms] OR “cytokines” [All Fields] OR “cytokine” [All Fields] OR “cytokinic” [All Fields] OR “cytokins” [All Fields])).

#### 2.2.3. Vitamin D

(“vitamin d” [MeSH Terms] OR “vitamin d” [All Fields] OR “ergocalciferols” [MeSH Terms] OR “ergocalciferols” [All Fields] OR (“ergocalciferols “[MeSH Terms] OR” ergocalciferols “[All Fields] OR” ergocalciferol “[All Fields]) OR (“ cholecalciferol “[MeSH Terms] OR” cholecalciferol “[All Fields] OR” cholecalciferols “[All Fields] OR” colecalciferol “[All Fields]) OR (“ calcitriol “[MeSH Terms] OR” calcitriol “[All Fields] OR” calcitriols “[All Fields])) AND (“ inflammation “[MeSH Terms] OR” inflammation “[All Fields] OR “inflammations” [All Fields] OR “inflammation s” [All Fields] OR (“inflammatories” [All Fields] OR “inflammatory” [All Fields]) OR “TNF” [All Fields] OR (“interleukine” [All Fields] OR “interleukines” [All Fields] OR “interleukins” [MeSH Terms] OR “interleukins” [All Fi elds] OR “interleukin” [All Fields]) OR (“cytokin” [All Fields] OR “cytokine s” [All Fields] OR “cytokines” [MeSH Terms] OR “cytokines” [All Fields] OR “cytokine” [ All Fields] OR “cytokinic” [All Fields] OR “cytokins” [All Fields])).

#### 2.2.4. Zinc

(“zinc” [MeSH Terms] OR “zinc” [All Fields]) AND (“inflammation” [MeSH Terms] OR “inflammation” [All Fields] OR “inflammations” [All Fields] OR “inflammation s” [All Fields] OR (“inflammatories” [All Fields] OR “inflammatory” [All Fields]) OR “TNF” [All Fields] OR (“interleukine” [All Fields] OR “ interleukines “[All Fields] OR” interleukins “[MeSH Terms] OR” interleukins “[All Fields] OR” interleukin “[All Fields]) OR (“ cytokin “[All Fields] OR” cytokine s “[All Fields] OR “cytokines” [MeSH Terms] OR “cytokines” [All Fields] OR “cytokine” [All Fields] OR “cytokinic” [All Fields] OR “cytokins” [All Fields])).

Mesh terms were not used to search the CLSR.

### 2.3. Study Selection

Two reviewers (RMB and MLM) independently reviewed the titles, abstracts and full texts for their potential inclusion against the eligibility criteria. Any disagreement was resolved by discussion with a third reviewer (SC). In cases where information about a study’s eligibility was limited or incomplete (for instance, when only an abstract was accessible), the authors of the study were contacted to request the full text or further details.

### 2.4. Data Extraction, Coding and Analysis

**Figure 1:**
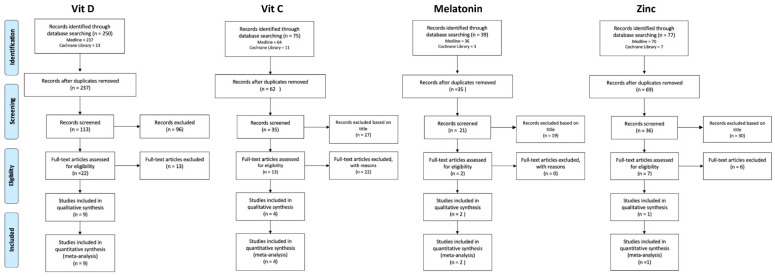
Flow diagram of the study selection process.

Two authors (MBR and LMM) collected data from all included articles using a pre-tested form and individuated duplicates, and prepared the flow-chart of excluded and included studies (Figure 1). SC and CA independently verified the entire process.

### 2.5. Quality Assessment of Included Reviews

**Figure 2:**
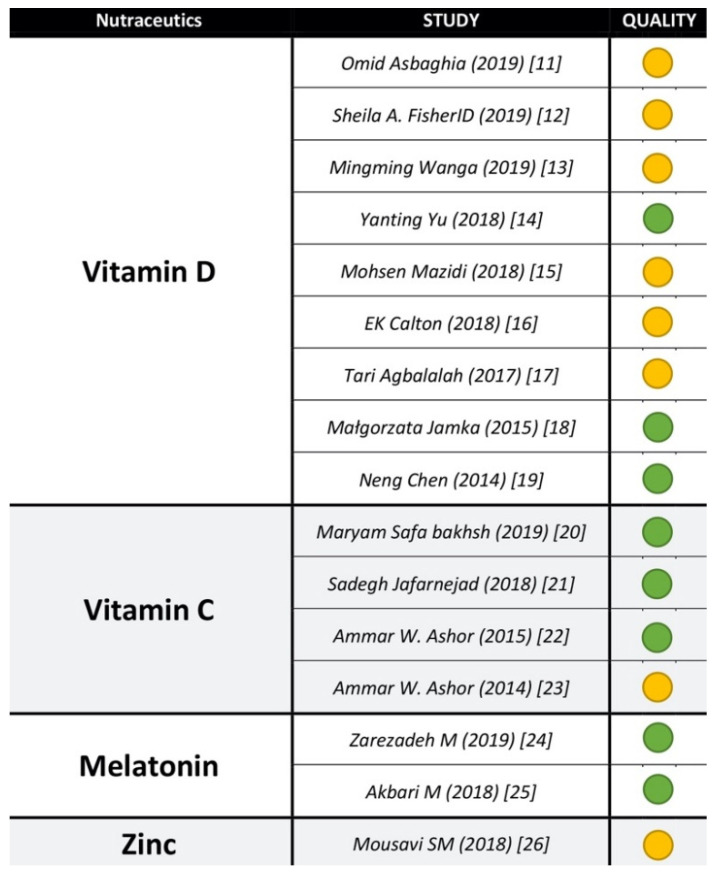
A Measurement Tool to Checklist Assess Systematic Reviews (AMSTAR) assessment for each systematic review, sorted by year of publication. Colors are referred to scores: green is referred to “high scores” (8–11 points) and yellow to “moderate” (4–7 points). No systematic review had a “low” (<3 points) evaluation.

Two authors (RMB and MLM) assessed the quality of the included systematic reviews by A Measurement Tool to Checklist Assess Systematic Reviews (AMSTAR). AMSTAR is a comprehensive validated tool to assess the methodological quality of SRs. It includes 11 domains, like the a priori protocol documentation, scientific quality and risk assessment publication bias. Based on AMSTAR evaluations, we ordered the AMSTAR scores into tertiles and classified the methodological quality of the single identified reviews in three categories: “high” (8–11 points out of 11), “moderate” (4–7 points) and “low” (3 or fewer points (Figure 2).

### 2.6. Dosage of Nutraceuticals

Data were extracted from each trial of the included systematic reviews/meta-analyses, and they were tabulated according to the results of each trial. We did not tabulate vitamin D dosage due to the great heterogeneity among trials.

## 3. Results

No SR regarding the four compounds was of low quality.

The punctual evaluation is reported in the Supplementary Materials.

Regarding vitamin D (Table 1), several SRs were taken into consideration, and they were heterogenous in different aspects: patient characteristics, type of treatment, end-points and measured variables.

However, this heterogeneity enriched the final analysis. Nine SRs were included, with a follow-up duration between 1,5 month and 3 years. The diabetic patients were mostly represented, but different categories of patients were considered, including those affected by HIV, obese, elderly or featuring a high cardiovascular risk. Six out of nine SRs demonstrated a clear efficacy of CRP reduction. Only one SR showed a reduction in interleukin-6 (IL6).

Table 2 shows the variable dosages used in each study, but an average of 50.000 units of ergocalciferol monthly were administered to patients.

Regarding vitamin C (Table 3), four SRs showed an effective action on the endothelial function and CRP reduction with an intervention duration between 1 day and 52 weeks. The diabetic patients, subjects with chronic diseases and adult participants aged 18 years and older were considered. Two out of four SRs proved to be effective in reducing CRP levels. The other two SRs showed beneficial effects on endothelial function.

One or two grams of vitamin C per day resulted in the most applicable posology to increase the efficacy of CRP reduction (Table 4).

Table 5 shows the characteristics of the included systematic reviews of melatonin. Two SRs showed an effective action of this hormone in terms of reduction in IL-6, TNF-alpha and CRP. The follow-up duration ranged between 4 months and 60 weeks. The two studies considered subjects with chronic diseases and individuals affected by metabolic syndrome.

From the analysis of the included trials, a variety of employed dosages emerge (Table 6); a daily dose from 5 to 25 mg daily has been reported, with similar efficacy.

Finally, we found only one SR of zinc supplementation summarizing 8 RCTs (Table 7). The intervention duration ranged between 6 and 25 weeks. The hemodialysis patients were considered.

Three RCTs had a positive effect on CRP reduction using 50 mg of elemental zinc daily (Table 8).

## 4. Discussion

Pathophysiological basis of nutraceutical supplementation during the COVID-19 pandemic.

### 4.1. Vitamin D

Recently, the use of cholecalciferol was proposed as a beneficial measure to reduce the risk of COVID-19, in order to manage the pro-inflammatory milieu [47], and to reduce virus-induced iron dysmetabolism [48]. Literature data show a scenario of a world epidemic of cholecalciferol deficiency [49], which reaches 80% in elderly people, who have been shown to suffer from a higher COVID-19 mortality rate [50]. The intensity of the host immune/inflammatory response has repercussions on the clinical severity and mortality risk associated with viral diseases such as COVID-19, and this factor could be influenced by vitamin D deficiency. Due to the low (20%) supply of cholecalciferol through dietary consumption, the supplementation of this pro-hormone has been suggested as beneficial in most chronic degenerative diseases. Specifically, active form calcitriol, a secosteroid hormone, can exert immuno-modulatory/anti-inflammatory activities, playing a role in the regulation of both innate and adaptive immunity; hence, in the COVID-19-related so-called “Cytokine storm”, it is considered to be caused by the activation of the innate immune system and with an excessively increased activation of the adaptive immunity [51,52]. With regard to innate immunity, calcitriol can inhibit inflammatory T cell cytokines and Toll-like receptors present on monocytes. Moreover, high doses of calcitriol supplementation result in a significant reduction in IL-6 [53]. With regard to adaptive immunity, vitamin D also reduces excessive proliferation and immunoglobulin production in B cells; furthermore, it may suppress the differentiation of B cells into plasma cells [54]. Cholecalciferol can reduce the risk of the common viral influenza enhancing the epithelium physical barrier mechanism, as well as through the modulation of native and adaptive immunity [55]. In addition, vitamin D is related to the preservation of adhesion junctions, tight and gap junctions between epithelial cells, and their destruction represents the pathogenic mechanism of viral upper respiratory tract infection [56,57]. Concerning COVID-19, it has been hypothesized that the correction of vitamin deficiency suppresses CD26/DDP4, one of the adhesion molecules through which the COVID-19 virus and COVID-MERS virus enter into host cells [58,59,60]. Calcitriol could also directly affect SARS-CoV-2 infection through the anti-microbial cathelicidin family of peptides, particularly LL-37 [18]. This peptide may induce a viral membrane disruption via electrostatic interactions on the lipid envelopes of viruses [18,19,61,62]. Cardiovascular thrombotic events have been associated with the later stages of COVID-19, together with a high prevalence of pulmonary embolism, disseminated intravascular coagulation, liver, myocardial and renal failure [63,64]. Calcitriol was proven to exert some anticoagulant effects by upregulating the expression of anticoagulant thrombomodulin and downregulating the expression of critical coagulation factor in monocytes [65,66,67]. This virus-induced prothrombotic state is worsened in the case of heme release in the blood stream, which results in an upregulation of NLRP3 inflammasome [68,69]. Finally, it can enhance the expression of antioxidant-related genes [26], and increase the production of glutathione, thereby avoiding the use of ascorbic acid (vitamin C), which results in an antimicrobial activity [70,71,72], reducing the production of free radicals involved in inflammation which contribute to the pulmonary damage leading to the development of acute respiratory distress syndrome (ARDS) [70]. Moreover, vitamin D plays a crucial role in the reduction in proinflammatory cytokines, such as TNFα and IFNγ, involved in the pathogenesis of ARDS through the stimulation of Th2 and inhibition of Th1 [73,74,75]. Recently, a retrospective study has evaluated the clinical outcomes of 36 out of 91 COVID-19 patients receiving in-hospital high-dose cholecalciferol. During a follow-up period of approximately two weeks, logistic regression statistical analysis indicated that the positive effect of high-dose cholecalciferol on the combined endpoint was significantly augmented with growing comorbidity burden [76]. Moreover, the intermediate form of vitamin D Calcifediol was proposed as an additional treatment in COVID-19 patients, as it could significantly reduce the need for ICU treatment [77]. A recent systematic review and meta-analysis showed that low vitamin D serum levels were significantly associated with a higher risk of COVID-19 infection [78]. Regarding the severity of the disease, another systematic review highlights that subjects affected by severe COVID-19 present 65% more vitamin D deficiency in comparison to individuals affected by mild COVID-19. In addition, vitamin D deficiency was related to increased hospitalization and mortality from COVID-19 [79].

### 4.2. Vitamin C

The biomedical literature supports the role of ascorbic acid (AA) (vitamin C) in immunity regulation, anti-infective and anti-NLRP3 (namely, cytokine storm) activity [80,81,82,83,84]. In fact, this essential compound intervenes in a number of fundamental biochemical pathways of cell metabolism, including mitochondria functionality. These organelles undergo a high degree of oxidative stress and mitophagy during COVID-19 degenerative processes [85]. Vitamin C was shown to prevent mitochondrial membrane depolarization and to combat mitochondrial DNA oxidative stress and cell toxicity, thus regulating fission and fusion processes [86,87]. Of interest, in the context of COVID-19-related hypoxia, AA interacts with hemoglobin to maintain heme iron in ferrous state, which is the only form to bind oxygen [88]. This exclusive beneficial activity on hemoglobin metabolism is best achieved by combining the supplementation of oral (L-AA) and intravenous AA [48,89,90] Overall, clinical data have previously highlighted a significant role for AA among patients in ICU, with sepsis, pneumonia, multiorgan failure and ARDS [91]. Similarly, vitamin C immunomodulatory, anti-viral and anti-inflammatory properties have been repeatedly demonstrated in infective diseases [72,80,92,93]. The combination of these beneficial biochemical features has led several centers to assess high-dose AA supplementation as a complementary therapy in COVID-19 patients [91,94,95]. A few preliminary clinical studies on vitamin C in critical COVID-19 patients have shown some improvements in the oxygenation and interleukin-6 level, though a lower benefit for the mortality rate has been reported [96,97,98]. Conversely, a more pronounced effect of vitamin C in combination with quercetin and bromelain seems to be effective, in terms of the prevention of COVID-19 infection in health-workers [99]. Ongoing clinical trials will likely provide more evidence on the possible efficacy of vitamin C in COVID-19 patients. A randomized clinical trial is analyzing the efficacy and safety of high-dose vitamin C in combination with Chinese medicine in the treatment of moderate and severe COVID-19 [100]. An uncontrolled longitudinal study is attempting to evaluate the efficacy and safety of 10 g of vitamin C intravenously in addition to conventional therapy in hospitalized patients with COVID-19 [101].

### 4.3. Melatonin

Melatonin (N-acetyl-5-methoxytryptamine) is a multifunctional hormone which is secreted mostly by the pineal gland and maximally at nighttime; [102] its secretion is extremely high in infants and adolescents, much lower in the elderly. Basically, this molecule helps to regulate many other hormones and maintains the body’s circadian rhythm. Melatonin is significantly involved in the complex network of psycho-neuroendocrine immunology (PNEI), stress management and aging mechanisms [103]; furthermore, this compound interacts with cortisol and with a series of immunity and inflammasome pathways, which have been shown to derange in COVID-19 [104]. This molecule has been repeatedly considered a potentially useful compound in COVID-19 patients [48,105,106,107]. In fact, in these cases, it may reduce the documented extremely high mitochondria oxidative stress, namely, in lung cells [108], while conferring a general antioxidant action [42]. Melatonin was also recognized as a relevant modulator of innate and adaptive immune reactions [109] and specifically of the inflammasome NLRP3 [110,111], the latter being a hyperactivated pathway in COVID-19 patients, contributing to the so-called “cytokine storm”. This inflammasome downregulation also results in a reduction in pulmonary hypertension, which typically occurs in the critical stages during Sars-Cov-2 infection [112]. Lastly, melatonin is known to interact with CD147, a favorite Sars-Cov-2 cell receptor which is diffused in cell walls, erythrocyte and endothelium specifically. This specific feature has been regarded as a protective mechanism against a few pathologic pathways which may occur during COVID-19, such as hemoglobin denaturation, iron accumulation, hypoxia, cardiomyocytes injury and hypercoagulability [104,106,113]. Very preliminary clinical data correlate the better survival rate of COVID-19 intubated patients with melatonin exposure [114]; similarly, a small randomized clinical trial has shown a statistically significant improvement in clinical and instrumental findings in a group of patients treated additionally with melatonin, with a more rapid hospital discharge and return to baseline health [115]. A deregulation of tryptophan (precursor of melatonin) production has been demonstrated in COVID-19, which led a few authors to postulate the need of a greater supplementation with this hormone [116]. Of great interest, a recent multidrug repurposing study on 26,779 subjects affected by COVID-19 elucidated that higher melatonin levels were significantly associated with a 28% and 52% reduced likelihood of a SARS-CoV-2 infection in the general population and in African Americans, respectively [117]. The combination of the potential and demonstrated beneficial activities of melatonin, which is currently being investigated in a series of specific clinical trials, may pave the way to a greater employment of this compound in this pandemic [118].

### 4.4. Zinc

The transition metal zinc (Zn), after iron, is the second most abundant trace metal in the human body, and it is essential for multiple cellular functions, including the preservation of immune health, playing a critical role in antiviral immunity. Zn also acts as an anti-inflammatory agent and functions as an antioxidant, membrane stabilizer. Of interest, Zn deficiency can lead to immunodeficiency and severe lymphopenia, which is caused by a corresponding decrease in developing B cells in the bone marrow; furthermore, Zn potentiates a type-I Interferon (IFN) effect. Marked neutrophilia is detected in severe COVID-19 patients. Zn gluconate supplementation, inhibiting the NFkB-dependent transcription of inflammatory genes, is able to reduce airway neutrophil infiltration and TNF-α release. Zn supplementation may be able to reduce inflammatory cytokines (IL-6 and IL-1β), enhancing the protective type-I IFN response in COVID-19 [119]. Zn inadequacy and deficiency are predicted to affect about 30% of the world population, in particular in the elderly; hence, the protective role of zinc supplementation against infection in the elderly population has been proposed [120]. Zn-deficient individuals experience increased susceptibility to pathogens, as well as some degree of ageusia and anosmia, emerging symptoms in COVID-19 patients [121,122]. Interestingly, coronavirus RNA polymerase activity appears to be inhibited by zinc [123], which could confer this metal a role in preventing coronavirus entry into cells [124] and reducing coronavirus virulency [125]. To date, scarce and contrasting clinical data on Zn supplementation efficacy on COVID-19 are available and particularly in the outpatient setting. A retrospective study including 141 individuals affected by COVID-19 in the general practice setting showed that zinc in combination with low-dose hydroxychloroquine was associated with significantly fewer hospitalizations [126]. On the contrary, in a recent randomized clinical trial of ambulatory COVID-19 patients, the administration of high-dose zinc gluconate or zinc gluconate combined with ascorbic acid did not significantly reduce the durability of symptoms in comparison with standard of care as well as hospitalizations and deaths [127].

### 4.5. Evidence

Regarding vitamin D, our review highlights a wide range of SRs, with different dosages and in different populations. The follow-up was between 1.5 months and 3 years. Different groups of patients were considered, including patients affected by diabetes, HIV, obese, elderly or featuring a high cardiovascular risk. Considering that different dosages of vitamin D (Table 2) showed a similar efficacy in CRP, the intake of 50,000 IU/month seems to be the proper dosage in terms of advantage and efficacy; moreover, this dose is in agreement both with the suggested dosage to reduce inflammatory activation and with the recommendations that advise not to exceed 4000 IU/day, chronically. Regarding vitamin C, our review identified four high-quality SRs. The intervention duration ranged from 1 day to 52 weeks Different categories of patients were considered, including diabetic patients, subjects with chronic diseases and adult participants aged 18 years and older. The analysis of each single trial (Table 4) suggests that the effective dose is between 1 to 2 g per day both for CRP and endothelial function. Due to the impracticability of the intravenous administration within the community medicine policy, oral intake is recommended. With reference to melatonin, our review highlighted two SRs, both of high quality at AMSTAR evaluation. The follow-up duration ranged from 4 months to 60 weeks. The two studies considered subjects with chronic diseases and metabolic syndrome.

Melatonin seems to show good evidence of efficacy regarding the reduction in CRP, TNF and IL6, with a dosage ranging from 5 to 25 mg/day (Table 7). However, the proper daily dose should be tailored to the age and clinical conditions of the patent, in order to avoid possible adverse effects, such as drowsiness. Literature data on zinc show a lower strength of evidence, in comparison to the other compounds. We found only one SR with an intervention duration between 6 and 25 weeks. The hemodialysis patients were included. The analysis of this single study (Table 8) showed positive results in CRP. A dosage of 50 mg/day of elemental zinc supplementation was proposed to achieve an adequate efficacy.

## 5. Conclusions

According to the selected systematic reviews, vitamin C, vitamin D, melatonin and zinc nutraceuticals have anti-inflammatory actions. Hence, their large-scale utilization seems to represent a useful and viable approach during the COVID-19 pandemic. Adequate doses should be employed, following the most referenced literature data. Of importance, since no specific drug or other therapeutic or preventive measure has proven to be beneficial against the progression of COVID-19, this nutraceutical approach could have a role within a community-based medicine. However, it is to be highlighted that, to date, no systematic review has demonstrated a specific preventive effectiveness of these compounds in COVID-19, and many clinical trials are ongoing. In view of this missing evidence, further research is necessary and desirable to obtain more data on the employment of these natural molecules as a prevention and/or treatment in the COVID-19 pandemic. Other future clinical trials should be designed to assess the clinical benefits of each nutraceutical and/or of the combination of two or more of them. Nevertheless, it might be more useful to implement a therapeutic supplementation campaign for measuring the effects on the global population. Observational studies (a design before–after study or cluster quasi-experimental study), primarily focusing on outcomes such as hospital rates and mortality, would be useful to preliminarily assess the efficacy of these compounds. Overall, the possible support of the national health systems would be instrumental to guarantee an adequate provision of these nutraceuticals to the population. On the basis of the reported data in this review, public health systems, and subsequently the World Health Organization, could somehow take into account the possibility to complement current limited preventative measures/interventions with targeted nutraceutical large-scale administration.

## Figures and Tables

**Table 1:** Vitamin D: characteristics of the included systematic reviews.

Study	Omid Asbaghia (2019) [11]	Sheila A. FisherID (2019) [12]	Mingming Wanga (2019) [13]	Yanting Yu (2018) [14]	Mohsen Mazidi (2018) [15]	EK Calton (2018) [16]	Tari Agbalalah (2017) [17]	Małgorzata Jamka (2015) [18]	Neng Chen (2014) [19]
**Databases searched**	PubMed, Scopus, ISI Web of Science and Google Scholar	Central, Medline, ENBASE, PubMed and Web of Science	PubMed, EMBASE, and Cochrane Library	PubMed and the Cochrane Library	PubMed-Medline, SCOPUS, Google Scholar and Web of Science	SCOPUS and PubMed	Cochrane, PubMed and Medline	PubMed, Scopus, the Cochrane Library and EMBASE	PubMed, Web of Science, and Cochrane library
**Articles included**	8	8	14	13	24	9	29	13	10
**Type of patients analyzed**	Healthy subjects and patients with colorectal adenoma, type 2 diabetes mellitus, pregnancy, pregnancy with gestational diabetes and polycystic ovary syndrome	Patients with type1 diabetes, Addison’s disease, multiple sclerosis, asthma and healthy subjects	Patients with asthma	Patients with type 2 diabetes	Patients with obesity, type 2 diabetic, HIV-infected, non-diabetic chronic kidney disease chronic fatigue syndrome, non-alcoholic fatty liver disease and healthy pregnant.	Patients ≥ 60 years, overweight and obese, prediabetes, non-alcoholic fatty liver disease, myocardial infarction, isolated systolic hypertension, postmenopausal women.	Patients with type 2 and gestational diabetes mellitus/prediabetic, cardiovascular disease, chronic kidney disease and overweight/obese participants	Obese and overweight subjects	Healthy subjects and patients with type 2 diabetes, polycystic ovary syndrome women, obese adults, coronary artery disease patients
**Posology**	500 mg/d Ca citrate + 200 IU/day vit D 1000 mg/d Ca citrate + 50,000 IU/day vit D	650 IU/day vit D 2000 IU/day vit D 4000 IU/day vit D 14,000 IU/day vit D 4000 IU/monthly vit D 140,000 IU/monthly vit D	500 UI/day vit D	200 IU/day vit D 6.000 IU/day vit D 25.000 IU/day vit D 50.000 IU/week vit D	400 IU/day to 11200 IU/day vit D	200 IU/day to 11200 IU/day vit D	4000 IU/weeks vit D	1000 IU/day to 7000 IU/day vit D	400 IU/day to 7000 IU/day vit D
**Intervention duration range**	6 weeks–3 years	3–12 months	1,5–12 months	8–52 weeks	4 weeks–12 months	12–52 weeks	8–52 weeks	4–52 weeks	9–48 weeks
**Endpoint**	The effect of vitamin D–calcium co-supplementation on inflammatory biomarkers in adults	The effect of vitamin D supplementation in enhancing absolute T regulatory cells (Treg) numbers in patients with inflammatory or autoimmune disease.	To assess the correlations of vitamin D status with asthma- related respiratory outcomes.	To examine whether or not the supplementation of vitamin D exhibits anti-inflammatory benefits in T2DM subjects	To evaluate the effect of vitamin D supplementation on C-reactive protein (CRP)	Causal links between vitamin D status [25(OH)D] and systemic inflammation	The effects of vitamin D supplementation on endothelial function and inflammation in adults	The effect of supplementation with vitamin D on selected inflammatory biomarkers in overweight and obese subjects.	To evaluate the association of vitamin D supplementation with circulating hs-CRP levels.
**Result**	A significant reducing effect of vitamin D–calcium co-supplementation on serum CRP concentrations in comparison with placebo. No significant effect of joint calcium and supplementation with vitamin D on serum concentrations of IL-6 (WMD: −1.45, 95% CI: −5.31, 2.41 pg/mL, *p* = 0.46) and TNF-α (WMD: −0.79, 95% CI: −2.19, 0.61 pg/mL, *p* = 0.26).	Planned meta-analysis was not possible due to the heterogeneous nature of the studies. Nevertheless, in a trial of autoimmune disorders which measured the proportion of Tregs, a significant difference was reported, with a higher percentage of Tregs observed in the vitamin D group (at 12 weeks, mean 6.4% (SD 0.8%) (vitamin D) vs. 5.5% (1.0%) (placebo).	Vitamin D supplementation was associated with a protective effect of exacerbation in patients with vitamin D insufficiency (vitamin D < 30 ng/mL) (RR: 0.76 95%Cl (0.61–0.95)). It was also demonstrated an improvement of their FEV1% (FEV1% < 80%) (MD: 8.3 95%Cl (5.95–10.64).	Vitamin D supplementation significantly decreased the circulating hs-CRP concentration (standard mean differences, −0.45 [95% CI −0.77 to −0.14], *p* = 0.005). No significant effect of vitamin D supplementation on IL-6 and TNF-α plasma concentration.	The results indicated that the vitamin D supplementation significant decreased the hs-CRP level by 0.45 μg/mL, whereas the vitamin D supplementation did not influence the TNF-α and IL-6.	There was no effect on the weighted mean difference (WMD) of IL-6 [(WMD (95% confidence interval) = 0.1, (−0.166, 0.366) pg/mL, *p* = 0.462)] or C-reactive protein (CRP) [(WMD = −0.324, (−1.007, 0.359) mg/L, *p* = 0.352)].	No significant change in both endothelial and inflammatory markers (*p* > 0.05).	Vitamin D supplementation did not influence on CRP (std. mean differences −0.11; 95% CI −0.27–0.04; *p* = 0.15), TNF-α (std. mean differences −0.13; 95% CI −0.38–0.12; *p* = 0.31) and IL-6 concentrations (std. mean differences 0.1; 95% CI −0.43–0.63; *p* = 0.71).	Vitamin D supplementation significantly decreased the circulating hs-CRP level by 1.08 mg/L (95% CI, −2.13, −0.03), with the evidence of heterogeneity. Subgroup analysis suggested a higher reduction of 2.21 mg/L (95% CI, −3.50, −0.92) among participants with baseline hs-CRP level ≥5 mg/L.
Conclusions *	Vitamin D–calcium co-supplementation has beneficial effect on serum CRP concentrations. A beneficial effect was not seen for IL-6 and TNF-α concentrations.	Vitamin D supplementation may increase Treg/CD3 ratios in both healthy individuals and patients with autoimmune disorders and may increase Treg function.	Vitamin D supplementation reduced the rate of asthma exacerbation, especially in patients with vitamin D insufficiency.	In T2DM subjects, vitamin D supplementation is beneficial for the reduction in hs-CRP but does not have a significant influence on TNF-α and IL-6.	Vitamin D supplementation had no impact on serum CRP, IL10, and TNF-α, while significantly increased serum IL6.	Available high-quality RCTs did not support a beneficial effect of cholecalciferol on systemic IL-6 and CRP.	The use of vitamin D supplementation as a therapeutic or preventative measure for CVD is not supported by evidence.	Supplementation with vitamin D does not have a significant influence on changes in the concentration of selected inflammatory biomarkers in the obese and overweight subjects.	Vitamin D supplementation is beneficial for the reduction in circulating hs-CRP.

* Author’s conclusions are reported.

**Table 2:** Vitamin D: summary of the principal characteristics of the included systematic reviews.

Pharmaceutical Drug		Dose	Follow-Up	Efficacy Yes	Efficacy No	Study
Vitamin D1 e D2	Paricalcitol	400 IU day	3 months	CRP		Mohsen Mazidi (2018) [15]
	Ergocalciferol	50.000 IU/ month	12 weeks–6 months	CRP		Mohsen Mazidi (2018) [15]
Vitamin D3	Cholecalciferol	200–6.000 IU/day 25.000–50.000 IU/week	8–52 weeks	CRP	TNF-α e IL6	Yanting Yu (2018) [14]
		400 IU/day–11,200 IU/day	4 weeks–12 months	IL6	CRP, IL10 e TNF-α	Mohsen Mazidi (2018) [15]
		4000 IU/week	8 weeks		FMD *, CRP, IL-6 e TNF-α	Tari Agbalalah (2017) [17]
		4000 IU/day	24 weeks	hs-CRP **		Neng Chen (2014) [19]
		≤4000 IU/day	>12 weeks	CRP	TNF-α e IL6	Yanting Yu (2018) [14]

* FMD = flow-mediated dilation (endothelial function parameters). ** hs-CRP = circulating high-sensitivity C-reactive protein.

**Table 3:** Vitamin C: characteristics of the included systematic reviews.

Scheme 2019.	Maryam Safabakhsh (2019) [20]	Sedagh Jafamejad (2018) [21]	Ammar W. Ashor (2015) [22]	Ammar W. Ashor (2014) [23]
**Databases searched**	PubMed, Scopus, ISI Web of Science e Google Scholar	Scopus, Cochrane Library, PubMed and Google Scholar	MEDLINE, Embase, Cochrane Library and Scopus	Medline, Embase, Cochrane Library, and Scopus
**Articles included**	11	12	46	44
**Type of patients analyzed**	Diabetic subjects/Nonsmokers	Patients with chronic diseases	Adult participants >18 years	Adult participants
**Posology**	500 mg/day	250 mg/day–1 g/day	500–2000 mg/day	500 mg/day–1 g/day
**Intervention duration range**	1 day–8 weeks	4–24 weeks	4–52 weeks	1 day–8 weeks
**Endpoint**	The effect of vitamin C on reducing CRP or hs-CRP level.	The effects of supplementation with vitamin C on serum C-reactive Protein (CRP) levels.	The effects of antioxidant vitamins C and E supplementation on endothelial function.	The effect of supplementation with vitamin C on endothelial function.
**Results**	Vitamin C could decrease CRP levels relative to placebo group ([WMD] = −0.73 mg/L: 95% CI: −1.30 to −0.15, *p* = 0.013).	Supplementation with vitamin C significantly lowered CRP among trials.	Significant improvements in endothelial function were observed in trials supplementing with vitamin C (500–2000 mg/d) (SMD: 0·25, 95% CI 0·02, 0·49, P¼0·043)	A beneficial effect of vitamin C on endothelial function was found (SMD: 0.50, 95% CI: 0.34, 0.66, *p* < 0.001)
**Conclusions**	Vitamin C supplementation might have a significant effect only on CRP reduction.	Vitamin C supplementation reduces serum CRP levels.	Supplementation with vitamin C improves endothelial function.	Supplementation with vitamin C improved endothelial function.

**Table 4:** Vitamin C: summary of the principal characteristics of the included systematic reviews.

Administration	Dose	Follow-Up	Endpoint	Efficacy	Study
Intravenous	250 mg/day	8 weeks	CPR	Yes	Biniaz 2014 [24]
	300 mg/day	24 weeks	CPR	Yes	Attallah 2006 [25]
Oral	1 g/day	10 days	EF *	Yes	De Marchi 2012 [26]
	1 g/day	4 days	CRP	Yes	Colby 2011 [27]
	1 g/day	4 weeks	CRP	Yes	Modi 2014 [28]
	2 g/day	4 weeks	EF *	Yes	Antoniades 2004 [29] Tousoulis 2007 [30]

* Endothelial function (EF) measured by either forearm blood flow (FBF) or flow mediated dilation (FMD).

**Table 5:** Melatonin: characteristics of the included systematic reviews.

	Zarezadeh M (2019) [31]	Akbari M (2018) [32]
**Databases searched**	SCOPUS, PubMed, Cochrane Library, Embase, Google Scholar	Cochrane Library, EMBASE, PubMed, and Web of Science
**Articles included**	13	6
**Type of patients analyzed**	Patients with chronic diseases	Patients with metabolic syndrome
**Posology**	3 to 25 mg/day	6 to 10 mg/day
**Intervention duration range**	From 4 to 60 weeks	From 4 weeks to 14 months
**Endpoint**	To evaluate the effect of supplementation with melatonin on inflammatory biomarker levels	To evaluate the effect of supplementation with melatonin on inflammatory markers among subjects with MetS or related disorders.
**Results**	Melatonin supplementation significantly decreased TNF-α and IL-6 levels [(WMD = −2.24 pg/mL; 95% CI −3.45, −1.03; *p* < 0.001; *I*2 = 96.7%, Pheterogeneity < 0.001) and (WMD = −30.25 pg/mL; 95% CI −41.45, −19.06; *p* < 0.001, 2*I* = 99.0%; Pheterogeneity < 0.001)], respectively. The effect of melatonin on CRP levels was marginal.	Melatonin supplementation significantly reduced C-reactive protein (SMD = −1.80; 95% CI −3.27, −0.32; *p* = 0.01; *I*2: 95.2) and interleukin 6 (IL-6) concentrations (SMD= −2.02; 95% CI −3.57, −0.47; *p* = 0.01; *I*2: 91.2) among patients with MetS and related disorders; however, it did not affect TNF-α concentrations.
**Conclusions**	Melatonin supplementation significantly reduced TNF-α and IL-6 levels. The supplementation with melatonin improved the levels of TNF-α and IL-6 more efficiently in studies, which were conducted for ≥ 12 weeks and at a dosage ≥ 10 mg/day.	The promising effect of melatonin administration on reducing CRP and IL-6 among patients with metabolic syndrome and related disorders.

**Table 6:** Melatonin: summary of the principal characteristics of the included systematic reviews.

Administration	Dose	Follow-Up	Endpoint	Efficacy	Study
Oral	25 mg/day	26 weeks	TNF and IL-6	Yes	SanchezLopez A (2018) [33]
	20 mg/day	12 weeks	TNF	Yes	Lissoni P (1996) [34]
	10 mg/day	12 weeks 12 weeks 4 weeks	CPR	Yes	Raygan et al. (2017) [35] Pakravan (2017) [36] Javanmard (2016) [37]
		26 weeks 60 weeks 4 weeks	TNF and IL-6	Yes	Forest CM (2007) [38] Celinski et al. (2014) [39] Cichoz-Lach et al. (2010) [40]
	6 mg/day	6 weeks	TNF and IL-6	Yes	Mesri Alamdari (2015) [41]
	5 mg/day	52 weeks	CPR	Yes	Chojnacki C (2011) [42]

**Table 7:** Zinc: characteristics of the included systematic reviews.

	Mousavi SM (2018) [43]
**Databases searched**	PubMed, SCOPUS, and Google Scholar
**Articles included**	8
**Type of patients analyzed**	Hemodialysis patients
**Posology**	50 mg/day
**Intervention duration range**	6–25 weeks
**Endpoint**	Effect of supplementation with zinc on plasma CRP concentrations in adults
**Results**	The results of the meta-analysis displayed a significant reduction in circulating CRP levels (WMD: −1.68 mg/L; 95% CI: −2.4 to −0.9, *p* =< 0.001) following supplementation with zinc.
**Conclusions**	Supplementation with zinc markedly reduced plasma CRP concentration

**Table 8:** Dose finding according to single trial results for zinc.

Administration	Dose	Follow-Up	Endpoint	Efficacy	Study
Oral	50 mg/day elemental zinc (220 mg zinc sulfate)	6 weeks 8 weeks 8 weeks	CPR	Yes	Rashidi (2009) [44] Tabrizi (2011) [45] Jamilian (2016) [46]

## Data Availability

Not applicable.

## Supplementary Materials

The following are available online at https://www.mdpi.com/article/10.3390/nu13041261/s1, Assessment of each systematic review by the AMSTAR tool.
