# Supplementary material for: Does Evidence Exist to Blunt Inflammatory Response by Nutraceutical Supplementation during COVID-19 Pandemic? An Overview of Systematic Reviews of Vitamin D, Vitamin C, Melatonin, and Zinc
Source: Nutrients. 2021 Apr 12;13(4):1261. doi: 10.3390/nu13041261 (PMC8069903; doi:10.3390/nu13041261)
Supplement: Supplementary file 1 [file nutrients-13-01261-s001.pdf]

Assessment of each systematic review by the AMSTAR tool

**AMSTAR Vitamin D**	***Omid Asbaghia (2019)***	***Sheila A. FisherID (2019)***	***Mingming Wanga (2019)***	***Yanting Yu (2017)***	***Mohsen Mazidi (2018)***	***EK Calton (2018)***	***Tari Agbalalah (2017)***	***Małgorzata Jamka (2015)***	***Neng Chen (2014)***
1.A-priori design	Yes	Yes	Yes	No	Yes	Yes	ND	Yes	ND
2.Duplicate study selection and data extraction	Yes	Yes	Yes	Yes	Yes	Yes	Yes	Yes	Yes
3.Comprehensive literature review	Yes	Yes	Yes	Yes	ND	Yes	Yes	Yes	Yes
*4.Unpublished grey reports Sought**	No	No	No	Yes	ND	ND	ND	Yes	Yes
5.List of included and excluded studies provided	No	No	No	No	NO	No	NO	No	No
6.Characteristics of individual studies provided	Yes	Yes	Yes	Yes	Yes	Yes	Yes	Yes	Yes
*7.Scientifi c quality of studies assessed and documented**	No	No	No	Yes	ND	No	No	No	Yes
8.Scientifi c quality of studies used for conclusions	No	ND	No	Yes	ND	No	No	ND	Yes
9.Statistical method appropriate	Yes	Yes	Yes	Yes	Yes	Yes	Yes	Yes	Yes
10.Likelihood of publication bias assessed	Yes	Yes	Yes	Yes	Yes	Yes	Yes	Yes	Yes
11.Conflict of interest declared in both systematic review and included studies	Yes	Yes	Yes	Yes	Yes	Yes	Yes	Yes	Yes
Aggregate score	7/11	7/11	7/11	9/11	5/11	7/11	6/11	8/11	8/11

**AMSTAR Vitamin C**	***Maryam Safa bakhsh (2019)***	***Sadegh Jafarnejad (2018)***	***Ammar W. Ashor (2015)***	***Ammar W. Ashor (2014)***
1.A-priori design	Yes	Yes	ND	ND
2.Duplicate study selection and data extraction	Yes	Yes	Yes	Yes
3.Comprehensive literature review	Yes	Yes	Yes	Yes
*4.Unpublished grey reports Sought**	No	Yes	No	No
5.List of included and excluded studies provided	No	No	No	No
6.Characteristics of individual studies provided	Yes	Yes	Yes	Yes
*7.Scientifi c quality of studies assessed and documented**	Yes	Yes	Yes	Yes
8.Scientifi c quality of studies used for conclusions	Yes	Yes	Yes	No
9.Statistical method appropriate	Yes	Yes	Yes	Yes
10.Likelihood of publication bias assessed	Yes	Yes	Yes	Yes
11.Conflict of interest declared in both systematic review and included studies	Yes	Yes	Yes	Yes
Aggregate score	9/11	10/11	8/11	7/11

**AMSTAR melatonin**	*Zarezadeh M (2019)*	*Akbari M (2018)*
1.A-priori design	Yes	Yes
2.Duplicate study selection and data extraction	Yes	Yes
3.Comprehensive literature review	Yes	Yes
4.Unpublished grey reports sought	No	No
5.List of included and excluded studies provided	No	No
6.Characteristics of individual studies provided	Yes	Yes
*7.Scientifi c quality of studies assessed and documented**	No	Yes
8.Scientifi c quality of studies used for conclusions	Yes	Yes
9.Statistical method appropriate	Yes	Yes
10.Likelihood of publication bias assessed	Yes	Yes
11.Conflict of interest declared in both systematic review and included studies	Yes	Yes
Aggregate score	8/11	9/11

**AMSTAR zinc**	***Mousavi SM (2018)***
1.A-priori design	ND
2.Duplicate study selection and data extraction	Yes
3.Comprehensive literature review	Yes
4.Unpublished grey reports sought	No
5.List of included and excluded studies provided	No
6.Characteristics of individual studies provided	Yes
*7.Scientifi c quality of studies assessed and documented**	No
8.Scientifi c quality of studies used for conclusions	Yes
9.Statistical method appropriate	Yes
10.Likelihood of publication bias assessed	
11.Conflict of interest declared in both systematic review and included studies	Yes
Aggregate score	6/11
